# Monocytic myeloid‐derived suppressor cells as prognostic factor in chronic myeloid leukaemia patients treated with dasatinib

**DOI:** 10.1111/jcmm.13326

**Published:** 2017-12-08

**Authors:** Cesarina Giallongo, Nunziatina L. Parrinello, Piera La Cava, Giuseppina Camiolo, Alessandra Romano, Marina Scalia, Fabio Stagno, Giuseppe A. Palumbo, Roberto Avola, Giovanni Li Volti, Daniele Tibullo, Francesco Di Raimondo

**Affiliations:** ^1^ Division of Hematology A.O.U. Policlinico‐OVE University of Catania Catania Italy; ^2^ Department of Biomedical and Biotechnological Sciences University of Catania Catania Italy

**Keywords:** myeloid‐derived suppressor cells, tyrosine kinase inhibitors, immunosurveillance, major molecular response, exosomes

## Abstract

Myeloid suppressor cells are a heterogeneous group of myeloid cells that are increased in patients with chronic myeloid leukaemia (CML) inducing T cell tolerance. In this study, we found that therapy with tyrosine kinase inhibitors (TKI) decreased the percentage of granulocytic MDSC, but only patients treated with dasatinib showed a significant reduction in the monocytic subset (M‐MDSC). Moreover, a positive correlation was observed between number of persistent M‐MDSC and the value of major molecular response in dasatinib‐treated patients. Serum and exosomes from patients with CML induced conversion of monocytes from healthy volunteers into immunosuppressive M‐MDSC, suggesting a bidirectional crosstalk between CML cells and MDSC. Overall, we identified M‐MDSC as prognostic factors in patients treated with dasatinib. It might be of interest to understand whether MDSC may be a candidate predictive markers of relapse risk following TKI discontinuation, suggesting their potential significance as practice of precision medicine.

## Introduction

Chronic myeloid leukaemia (CML) is a haematopoietic stem cell malignancy characterized by the t(9;22) chromosomal translocation that generates the BCR/ABL oncogene 1, 2. Therapy with tyrosine kinase inhibitors (TKI) has significantly improved patients survival 3. In addition, a subset of CML patients with a complete molecular response remain in remission after imatinib discontinuation although they still harbour a residual disease as indicated by deeper analyses (STOP‐IM) 4, 5. Recent reports have demonstrated that immunological diversity as well as genetic background could have a role in the efficacy and safety of imatinib cessation in patients with CML 6, 7. There is increasing evidence suggesting that increased NK cell counts seem to correlate with successful imatinib discontinuation; therefore, immunological surveillance may play an important role in these patients 8, 9. Current observations suggest a reciprocal interplay between tumour cells, stroma and the immune system that create an immunosuppressive microenvironment by releasing immunosuppressive molecules 10, 11, 12. CD34^+^ CML cells express programmed death receptor ligand 1 (PD‐L1) that suppress T cell functions by binding its receptor PD‐1 on T lymphocytes 11. Moreover, accumulation of regulatory T cells (Treg) 10 and myeloid‐derived suppressor cells (MDSC) has been described in patients with CML at diagnosis 13. MDSC are a heterogeneous group of immature myeloid cells that are dichotomized into granulocytic (G‐MDSC) and monocytic (M‐MDSC) subsets, identified by two different phenotypes: G‐MDSC are defined as CD11b^+^ CD33^+^ CD14^−^ HLA‐DR^−^ cells, while M‐MDSC are CD14^+^ HLA‐DR^−^ cells 14. Both subpopulations are able to inhibit T cells using different mechanisms, including up‐regulation of arginase 1 (ARG1), nitric oxidase synthase 2 (NOS2), reactive species of oxygen (ROS), cyclooxygenase 2 (COX2), transforming growth factor β (TGF‐β) and immunosuppressive cytokines 15. The phenotypical and functional diversity of MDSC could derive from the divergent composition of tumour‐derived factors that governs MDSC induction, expansion and activation, and that depends on cancer cell type 16. In addition, it has been demonstrated that CML G‐MDSC frequency correlates with the percentage of Treg in patients at diagnosis 12.

Recently, the prognostic role of MDSC accumulation has been documented for some haematological malignancies such as Hodgkin lymphoma, multiple myeloma and acute leukaemia patients, where they correlates with disease progression and persistence of minimal residual disease 17, 18. Contrary to these malignancies, CML is a myeloproliferative disorder characterized by the expansion of a clone of haematopoietic cells that carry BCR/ABL. Therefore, there is an important overlap between the tumour population and MDSC, as previously demonstrated 11, 12. This study focused on defining the change of MDSC frequency in CML patients during therapy with imatinib (IM), nilotinib (NIL) or dasatinib (DAS). We also evaluated the ability of serum from patients with CML and exosomes released from leukaemic cells to generate CD14^+^ HLA‐DR^−^ cells from healthy donor‐derived monocytes and ability of these cells to inhibit T cell proliferation. Our data suggest that CML cells are able to promote MDSC expansion creating an immunotolerant environment that results in T cell anergy and favours tumour growth.

## Materials and methods

### Patients and sample collection

This study has been approved by the local ethical committee (Azienda ospedaliero Universitaria Policlinico‐Vittorio Emanuele, #34/2013/VE). After written informed consent, samples were collected from patients with CML and age‐matched healthy donors (HD) at Division of Hematology, AOU Policlinico—OVE, University of Catania. This study enrolled 59 patients with CML, and for 42 of them, samples were collected at diagnosis too. Twenty patients were treated with IM (14 of whom evaluated also at diagnosis), 20 with NIL (15 evaluated at diagnosis) and 19 with DAS (13 evaluated at diagnosis). Among NIL‐treated patients, six were in second‐line treatment, while for DAS, five patients were in second‐line and one in third‐line treatment (all six patients changed TKI because of IM resistance). During treatment, all patients were followed with a monthly CBC count, molecular evaluation of the BCR/ABL transcript every 3 months and cytogenetic evaluation every 6 months, according to ELN guidelines. Clinical data of patients with CML at diagnosis included in this study are shown in Table 1.

**Table 1:** Clinical disease characteristics of patients

Patients	Gender	Age	BCR/ABL transcript levels	HGB (g/dl)	WBC (10^3^/μl)	PLT (10^3^/μl)	LDH (mg/dl)	Liver (cm)	Spleen (cm)	Blast count	Sokal score	HASFORD score	M‐MDSC (%)	Gr‐MDSCs (%)
1	M	67	81.37	11.2	72.2	355	2087	0	0	0	Low	Int	5.3	86.9
2	M	77	105.84	14.2	54.6	391	1426	0	0	0	Intermediate	Low	31	85
3	F	73	59.77	11.1	30.7	651	–	0	0	0	Low	–	0.7	72.7
4	M	56	66.29	12	164	526	2418	2	0	0	High	Intermediate	20.02	82
5	F	69	58.002	12.1	34	607	–	0	0	0	Intermediate	Low	10.2	81.4
6	M	84	45.1	14	50.3	285	873	0	0	0	Intermediate	Intermediate	23	79
7	M	51	41.59	12.5	38.4	368	–	0	0	0	Low	Low	26.9	87
8	F	56	20.41	12.6	95	463	668	0	–	0	Intermediate	–	29.8	79.4
9	M	62	79.85	11.3	331	96	–	6	4	5	High	Intermediate	37.7	85.4
10	M	59	39.74	12.4	152.24	527	–	2	3,5	0	Intermediate	–	50	88.7
11	M	59	25.14	15.3	22.8	20	–	0	0	0	Low	Intermediate	18.9	87
12	F	48	81.88	8.1	288	458	1043	2	14	3	High	Intermediate	41	86
13	F	71	48.16	9.2	70.5	338	2230	–	–	–	Intermediate	Intermediate	46.8	89
14	M	70	349.51	13.5	71.2	370	715	0	0	1	Intermediate	Intermediate	81.2	79
15	F	66	126.73	12.9	68.4	294	888	2	3	0	Intermediate	Intermediate	12.4	82
16	M	38	65.61	13.6	28.4	232	–	0	0	0	Low	Low	28.5	83.4
17	M	54	74.22	12.7	58	201	–	–	–	–	Low	Low	14.7	82
18	M	21	126.51	14.2	144	107	1247	0	6	1	Low	Low	81.6	83.7
19	M	53	142.78	16.6	34	311	288	0	3	0	Low	Intermediate	63	83
20	M	61	33.66	14.4	51.6	399	983	0	0	1	Low	Low	25	81
21	M	48	40.33	9.9	256	350	1074	7	14	1	Intermediate	Low	41	90
22	F	64	28.21	10.6	128.4	531	1147	0	2	1	Intermediate	Intermediate	1	58.3
23	F	57	122.97	11.4	156.6	273	1124	2	1	1	Low	Intermediate	61	88
24	M	36	56.24	10.8	55	208	1352	7	8	1	Low	Low	42.4	87
25	M	52	14.89	10.2	46	418	1820	0	2	1	Low	Intermediate	25	78
26	F	58	150.04	10.7	122.5	361	1683	0	3	1	Intermediate	Intermediate	50	75
27	F	65	191.66	15.3	87	252	688	0	0	0	Low	Low	91.2	50
28	F	72	71.98	13	111	168	345	4	2	2	Intermediate	Intermediate	14.4	75
29	F	78	48.26	11.7	77	651	–	0	1	2	High	Intermediate	5.4	64
30	M	37	153.59	12.8	91.6	344	1635	0	0	0	Low	Low	2.8	82.5
31	M	60	47	11	70	368	873	0	0	0	Intermediate	Intermediate	28	88
32	M	53	23.8	13	44	343	1820	0	2	1	Low	Intermediate	25	79
33	F	62	31.5	11	22	98	–	0	0	0	Low	Low	25.8	72
34	F	73	68.8	14	315	543	723	6	4	5	High	Intermediate	55	75
35	F	67	58.7	12.6	120	521	–	2	2	0	Intermediate	–	50	83
36	M	58	23.00	12.4	23	44	–	0	0	0	Low	Intermediate	16	87
37	M	47	78.2	11.9	98	98	1043	2	0	3	High	Intermediate	46	86
38	M	47	63	13.3	195	345	668	0	2	0	Intermediate	–	44	86
39	M	55	21	12.1	54	430	–	0	0	0	Intermediate	Low	11	78
40	F	63	120	10.7	70	370	1683	2	3	1	Intermediate	Intermediate	50	81
41	M	43	44.6	12	122	333	2230	–	–	–	Intermediate	Intermediate	37	77
42	M	69	328	9	71	370	715	0	0	1	Intermediate	Intermediate	83.2	84

The frequency and the functional characteristics of MDSC analysed in the PB from patients with CML at diagnosis. HD were age‐matched. (F, female; M, male; HGB, haemoglobin; WBC, white blood cells; PLT, platelets; LDH, lactate dehydrogenase. BCR/ABL transcipt levels are calculated as BCR‐ABL/ABL).

### Flow cytometry analysis of MDSC phenotype

The amount of MDSC was evaluated in peripheral blood (PB). Analysis of MDSC was performed with multicolor FACS analysis using the following antibody (Beckman Coulter, Beckman Coulter s.r.l. Milan, Italy): CD14 PC5 (clone RMO52), HLA‐DR ECD (clone IMMU‐357), CD11b FITC (clone bear‐1), CD33 PE (clone D3HL60, 251) and their respective isotype controls. Briefly, 1 × 10^6^ cells were stained with 10 μl of each of the above‐listed Abs and incubated for 20 min. in the dark at room temperature. After lysing red cells with ammonium chloride, cells were analysed by flow cytometer (Cytomics FC 500, Beckman Coulter), and analysis was performed with CXP Analysis software. Using sequential gating strategy (Supplementary material), G‐MDSC cells were identified as cells CD11b^+^CD33^+^CD14^−^HLA‐DR^−^, while the M‐MDSC as CD14^+^HLA‐DR^−^. The results were expressed as percentage.

### Functional characterization of MDSC

To evaluate the suppressive ability, G‐MDSC and M‐MDSC from patients with CML and HD were first isolated using magnetic separation (CD66b‐positive selection for G‐MDSC and CD14‐positive/HLA‐DR‐negative for M‐MDSC, Miltenyi Biotec, GmbH, Bergisch Gladbach, Germany), and then the purity and viability were tested by flow cytometry; viability was more than 90%. MDSC were cocultured for 3 days with autologous carboxyfluorescein succinimidyl ester (CFSE)‐labelled T lymphocytes at ratio 1:4 12. For cell labelling, 5 × 10^5^ lymphocytes were incubated at 37°C for 20 min. in 1 ml PBS containing 1 μM CFSE (BD Pharmingen). T cells were stimulated with 5 mg/ml phytohaemagglutinin (PHA) and incubated for 72 hrs prior to flow cytometry. Controls included a positive T cell proliferation control (T cells plus PHA) and a negative one (T cells only). After 3 days, T cell proliferation was measured by CFSE dilution and analysed using flow cytometry.

### Western Blot analysis

Western Blot analysis was performed according to the manufacturer's recommendations. The antibodies directed against the human Tsg101 and CD63 were obtained from Santa Cruz Biotechnology. The blots were scanned and determined using Scion Image software (New York, NY).

### Soluble factors and exosomes for the generation of M‐MDSC

Purification of monocytes from PB of 4 HD was performed with a positive selection of these cells using a magnetic separation kit (EasySep, STEMCELL Technologies, Vancouver, BC, Canada). Cell purity was determined by flow cytometry and was >90%. Monocytes were cultured with RPMI‐1640 medium with 1% penicillin–streptomycin supplemented with 20% FBS or HD (*n* = 4) or CML sera (*n* = 6). After 72 hrs of incubation, cells were stained with M‐MDSC Abs for flow cytometry analysis.

HD monocytes were also cultured in the presence of exosomes (30 μg protein/10^6^ monocytes) isolated from five CML serum patients at diagnosis.

### Isolation of serum exosomes

Serum exosomes were isolated and purified by differential ultracentrifugation according to a standard protocol for isolation of exosomes from viscous bodily fluids 19. Serum was derived from heparinized blood, diluted 1:2 with PBS (phosphate‐buffered saline) and centrifuged for 30 min. at 2000 × *g* at 4°C. The supernatant was transferred to ultracentrifuge tubes and centrifuged using a 13.1 JS rotor (Beckman Instruments, Inc., Fullerton, CA) for 30 min. at 12,000 × *g*, 4°C. Supernatant was carefully transferred into fresh ultracentrifuge tubes and centrifuged using a SW28 rotor (Beckman Instruments, Inc.) at 110,000 × *g* for 2 hrs at 4°C. The resulting pellet, resuspended in 1 ml of PBS, was diluted with PBS, filtered through a 0.22‐μm filter (Millex GP filter unit, Millipore, Billerica, MA) into fresh ultracentrifuge tubes and centrifuged in a SW28 rotor at 110,000 × *g* for 70 min. at 4°C. Then the tube containing the pellet was resuspended in 1 ml of PBS, filled with PBS and centrifuged at 110,000 × *g* for 70 min. at 4°C. The crude exosomes were resuspended in 50–100 μl of PBS for their characterization by scanning transmission and immunoelectron microscopy.

### Scanning electron microscopy (S.E.M.)

Exosomes (20 μl) were fixed in 80 μl of 2% glutaraldehyde–0.1 M phosphate buffer and fixed overnight at 4°C. A drop of suspension was layered on a sterile cover glass coated with 0.1% poly‐L‐Lysine, post‐fixed in 1% osmium tetroxide (Merck, Darmstadt, Germany) in the same buffer for 1 hr at 4°C and washed in phosphate buffer. After dehydrating in a graded ethanol and critical point drying, the samples were sputtered with a 5 nm gold layer using an Emscope SM 300 (Emscope Laboratories, Ashford, UK) and then observed. A Hitachi S‐4000 (Hitachi High‐Technologies America, Inc., Schaumburg, IL) field emission scanning electron microscope was used for the observations.

### Transmission electron microscopy (TEM)

Exosomes (20 μl) were fixed in 80 μl of 3% formaldehyde–0.1% glutaraldehyde overnight at 4°C. Five microlitres of the above suspension was layered on a formvar copper‐coated nickel grids (Electron Microscopy Sciences, Fort Washington, PA) and allowed to dry for 20 min. to absorb exosomes. The grids, washed in PBS, were negatively stained with 4% uranyl acetate for 5 min. and viewed using a Hitachi H‐7000 transmission electron microscope (Hitachi High‐Technologies Europe GmbH, Krefeld, Germany). For immunogold labelling, the grids were rinsed for 2 × 2 min. with PBS and transferred in a TBS (Tris‐buffered saline pH 7.4) solution containing 1% BSA (bovine serum albumin) (TBS/BSA) for 10 min. at room temperature. Then the grids were incubated in blocking solution 5% BSA for 1.30 hr at room temperature, rinsed with PBS and incubated in a humid chamber overnight at 4°C with a mouse monoclonal antibody CD81 (Santa Cruz Biotechnology, Heidelberg, Germany) in a dilution 1:50 with TBS/BSA. After wash for 3 × 3 min. with TBS/BSA, the grids were stained with a 10 nm gold‐labelled secondary antibody antimouse IgG (Sigma‐Aldrich, S.r.l., Milan, Italy) in a dilution 1:5 with TBS/BSA at 37°C for 1 hr in the dark. The grids were rinsed 2 × 2 with TBS/BSA, 2 × 2 with water and fixed with 1.5% glutaraldehyde in PBS for 10 min. at room temperature. After rinsed again with water, the grids were post‐stained with 4% uranyl acetate for 5 min. and allowed to air‐drying. Observations were carried out using the transmission electron microscope. Negative controls were prepared in the absence of primary antibody but with secondary antibody conjugate.

### Statistical analysis

The data are expressed as mean ± S.D. Statistical analysis was carried out by paired Student's *t*‐test, ANOVA test or Mann–Whitney *U*‐test. For correlation analysis, the Pearson's correlation was assessed. A *P* value <0.05 was considered to indicate a statistically significant difference between experimental and control groups.

## Results

### Increased levels of circulating MDSC in CML patients at diagnosis

**Figure 1:**
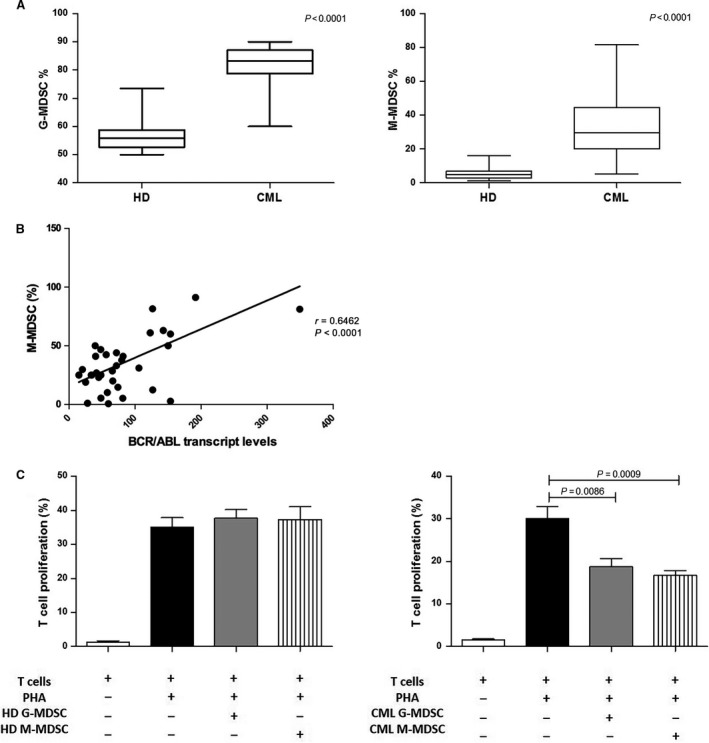
Increased frequency of MDSC in untreated CML patients. (**A**) The percentages of circulating G‐MDSC and M‐MDSC were quantified in the peripheral blood of HD and newly diagnosed patients with CML by flow cytometry. Flow cytometry analysis was performed with gates set on either CD11b^+^ CD33^+^ CD14^−^ HLA‐DR ^−^ (G‐MDSC) or CD14^+^ HLA‐DR ^−^ (M‐MDSC) cell populations. The bars represent the standard error of the mean. (**B**) Correlation analyses of the peripheral M‐MDSC count with BCR/ABL transcript levels calculated using the Pearson's correlation analysis. (**C**) Granulocytic and monocytic MDSC‐mediated T cell suppression in autologous cocultures. MDSC was previously tested for cell viability using cytofluorimetric analysis. Mean frequency of CD3^+^ CFSE ^dim^ cells ± S.D. from four independent experiments in duplicate is shown.

G‐MDSC and M‐MDSC percentages in patients with CML at diagnosis were greater than HD volunteers (84 ± 9% *versus* 56.2 ± 5.4% and 32 ± 20% *versus* 5.9 ± 4%, *P* < 0.0001, respectively) (Fig. 1A). Moreover, the frequency of M‐MDSC significantly correlated with BCR/ABL transcript levels (*r* = 0.64, *P* < 0.0001) (Fig. 1B). The percentages of G‐MDSC and M‐MDSC did not correlate neither with age, nor with leukocytosis or Sokal risk.

To validate whether these increased myeloid subpopulations were MDSC cells, their immunosuppressive activity was investigated. For this purpose, we isolated by magnetic separation CD14‐negative (representative of M‐MDSC) and CD66b‐positive (representative of G‐MDSC) cells from both CML patients at diagnosis and HD and incubated them with autologous CFSE‐labelled T cells. We found that CML G‐MDSC and M‐MDSC were able to inhibit T cell proliferation in comparison with positive control (from 30 ± 4.8% to 18.7 ± 3.8% for G‐MDSC, *P* = 0.0086 and to 16.7 ± 2.5% for M‐MDSC, *P* = 0.0009; Fig. 1C). In contrast, no suppressive effect was observed incubating G‐MDSC and M‐MDSC obtained from HD with autologous T lymphocytes.

### The percentage of M‐MDSC correlates with MMR in patients treated with dasatinb

**Figure 2:**
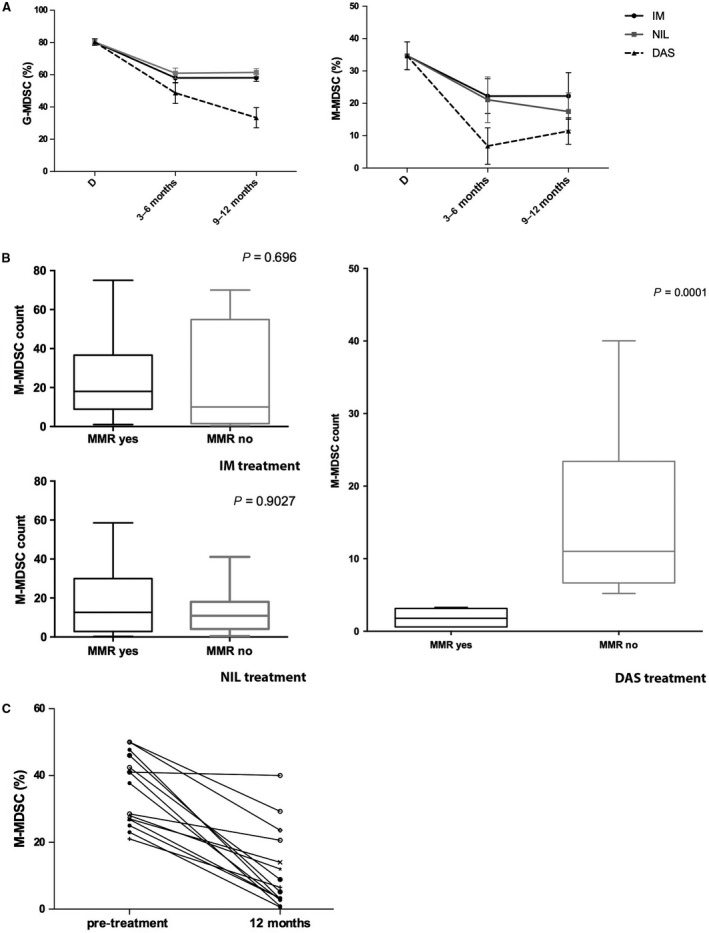
MDSC after TKI therapy. (**A**) Changes in circulating G‐MDSC and M‐MDSC in CML patients treated with IM, NIL or DAS. The bars represent the standard error of the mean. G‐MDSC at 3–6 and 9–12 months after IM, NIL and DAS:* P* < 0.0001. M‐MDSC after 3–6 months of DAS therapy: *P* < 0.05; after 9–12 months of DAS treatment: *P* < 0.01. (**B**) The percentage of M‐MDSC was compared between the MMR and no MMR groups. The bars represent the standard error of the mean. Statistical difference was calculated using Mann–Whitney *U*‐test. (**C**) M‐MDSC count for patients at diagnosis and after 12 months of therapy with DAS. Lines with empty circle represent patients no in MMR at 12 months. MMR, major molecular response; BCR‐ABL is ≤0.1%.

To explore the effect of TKI treatment, the percentages of G‐MDSC and M‐MDSC were evaluated during therapy with IM, NIL or DAS. As shown in Figure 2A, we found that both IM, NIL and DAS induced a significant reduction in G‐MDSC at 3–6 months (from 82.5 ± 9.6% to 55 ± 17.3% after IM, to 60.9 ± 9% after NIL and to 48.7 ± 13% after DAS, *P* < 0.0001) and 9–12 months (64 ± 8% after IM, 61 ± 6.3% after NIL and 32 ± 15% after DAS, *P* < 0.0001) of treatment. On the contrary, the frequency of M‐MDSC significantly decreased after DAS therapy only (from 33.6 ± 19% to 6.8 ± 12.6% at 6 months, *P* = 0.014 and to 12 ± 11.8% at 12 months, *P* = 0.004). M‐MDSC reduction was also present but did not reach statistical significance after IM treatment (22.2 ± 24.5% and 20.8 ± 18.6%, respectively, at 6 and 12 months) and after NIL therapy (21 ± 19.9% and 19 ± 17% at 6 and 12 months) with a great variability among patients.

As MDSC accumulation correlates with disease progression and minimal residual disease in myeloma and leukaemia patients 17, 18, we investigated the correlation of MDSC with clinical response to TKI therapy. We found that in DAS, but not in IM‐ and NIL‐treated patients (Fig. 2B), there was a correlation between major molecular response (MMR) values and number of persistent M‐MDSC at 12 months. Indeed, a significant difference was observed comparing the percentage of M‐MDSC in the MMR group (*n* = 8) *versus* no MMR (*n* = 11) (*P* = 0.0025). M‐MDSC count for patients evaluated both at diagnosis and after 12 months of dasatinib treatment is shown in Figure 2C.

Nevertheless, as for IM‐ and NIL‐treated patients, no correlation was observed between the levels of M‐MDSC at diagnosis and the response‐based therapy.

### CML cells induce M‐MDSC by secreting soluble factors

**Figure 3:**
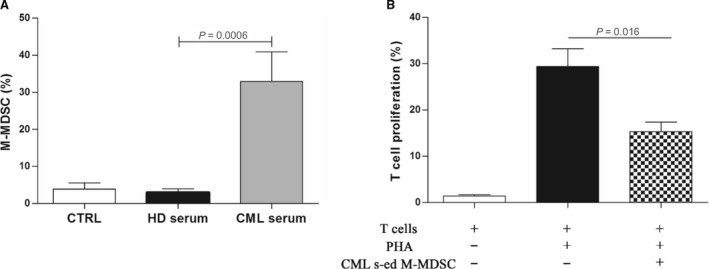
CML serum induces M‐MDSC with T cell suppressive ability. (**A**) Monocytes displayed phenotypic conversion into CD14^+^ HLA‐DR ^−^ after incubation with CML serum for 3 days. Results represent the means of four independent experiment; error bars denote S.D. (**B**) Suppressive activity of CML serum‐educated M‐MDSC (CML s‐ed M‐MDSC) was evaluated in coculture experiments with CFSE‐labelled autologous T lymphocytes. Mean frequency of CD3^+^ CFSE ^dim^ cells ± S.D. from four independent experiments in duplicate is shown.

To evaluate whether leukaemic cells were able to expand MDSC by releasing soluble factors, we incubated monocytes obtained from HD with sera from healthy volunteers or CML patients at diagnosis. Monocytes displayed phenotypic conversion into CD14^+^HLA‐DR^−^ only in conditions with CML serum; the percentage of M‐MDSC significantly increased by 29 ± 13%, *P* = 0.0006 (Fig. 3A). No increase in M‐MDSC frequency was found by incubating monocytes with serum from HD. On the contrary, G‐MDSC percentage was not increased by addition of CML or HD serum. In line with their MDSC‐like phenotype, CML serum‐educated monocytes showed suppressive ability after magnetic isolation and subsequent incubation with autologous T lymphocytes (Fig. 3B).

### CML‐derived exosomes promote M‐MDSC expansion

**Figure 4:**
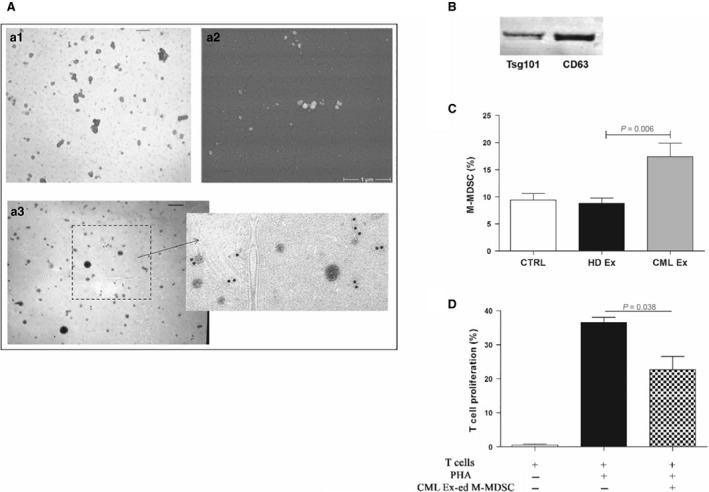
CML exosomes promote the generation of M‐MDSC. (**A**) a1: Representative TEM image of CML serum exosomes (Ex). The exosomes show a characteristic ‘deflated football‐shaped’ of 60–100 nm in size (Bar = 120 nm). a2: A S.E.M. image of CML exosomes at high magnification (× 30,000). a3: The exosomes are positive for exosomal marker CD81 (Bar = 120 nm). Right panel: boxed area shown at higher magnification. (**B**) Western blot analysis of protein extracted from exosomes. (**C**) An increase in the percentage of CD14^+^/HLA‐DR ^−^ cells was observed *in vitro* after incubation of HD monocytes with CML exosomes (*P* < 0.05). Results represent the means of four independent experiment; error bars denote S.D. (**D**) Suppressive activity of CML exosomes‐educated M‐MDSC (CML Ex‐ed M‐MDSC) was evaluated in coculture experiments with CFSE‐labelled autologous T lymphocytes. Mean frequency of CD3^+^ CFSE ^dim^ cells ± S.D. from four independent experiments in duplicate is shown.

A number of studies have recently described tumour‐released exosomes as new players in modulating the tumour microenvironment, promoting angiogenesis and tumour development 20. Notably, exosomes derived from human tumours inhibit functions of immune cells 21. Purified exosomes isolated from serum of patients with CML satisfied three major criteria as exosomes: they had a size of 50–100 nm in diameter (Fig. 4a1–2) and a density of 1.13–1.21 g/dl in a sucrose gradient; were enriched with CD80 (Fig. 4a3) and expressed Tsg101 and CD63 proteins (Fig. 4B). Interestingly, HD monocytes acquired a M‐MDSC phenotype when incubated with CML exosomes (from 9.4 ± 2.7% in untreated cells to 17.4 ± 5.5% in CML exosomes‐treated monocytes; *P* = 0.006; Fig. 4C). Incubation of monocytes with HD‐derived exosomes did not induce generation of M‐MDSC or G‐MDSC. We also demonstrated the immunosuppressive activity of CML exosomes‐educated M‐MDSC by documenting their ability to reduce autologous T cell proliferation (Fig. 4D).

## Discussion

Expansion of occult malignant clones is prevented by the concerted action of innate and adaptive immune system. The development of an overt neoplastic disease, such as CML, requires an escape from immune recognition through a multifaceted process of immunoediting whereby tumour‐reactive cytotoxic cells are either deleted or rendered anergic 22. TKI therapy eradicates very efficiently the majority of leukaemic cells, but it is not able to kill the most primitive quiescent leukaemic stem cells (LSC) 23, 24, 25. Therefore, discontinuation of TKI therapy often causes rapid disease relapse, presumably due to the reactivation of dormant LSC. Nevertheless, accumulating evidence indicates that some CML patients can stop imatinib treatment without suffering disease relapse after achieving a complete molecular response (CMR) 26. Therefore, specific predictive markers are needed to determine which patients can discontinue treatment without experiencing relapse.

We have previously demonstrated that there is a significant accumulation of MDSC in patients with CML at diagnosis that exert immunosuppression through Arg1 release essentially by polymorphonuclear cells 12, confirming results obtained by Christiansson *et al*. 11. These authors investigated only G‐MDSC cells and found their phenotype (CD11b^+^CD14^−^CD33^+^) on both CD34^+^ and CD34^−^ cells in the peripheral blood, demonstrating that not only healthy myeloid cells but also a proportion of the leukaemic myeloid cells show a MDSC phenotype. We have confirmed this observation through demonstration that both granulocytes and monocytes express, at least in part, BCR/ABL. The current work focused on defining the effect of the TKI therapy on MDSC count, investigating possible correlations with clinical response. Imatinib, nilotinib and dasatinib significantly decreased the amount of G‐MDSC. However, reduction in monocytic MDSC subset was not significant during imatinib and nilotinib therapy and was efficiently reduced in dasatinib‐treated patients only. This difference may be due in part to down‐regulation of Src and NF‐KB signal cascades as demonstrated for the inhibition of Treg 27 and MDSC in head and neck cancer 28. Moreover, some studies reported that after dasatinib treatment, an immunostimulation can be observed with increased numbers of CD8^+^ T cells, NK cells and decreased numbers of Treg 29, 30. These effects seem to be dasatinib‐specific as they were not observed with imatinib, nilotinib or bosutinib treatment. Therefore, immunostimulatory ability of dasatinib may be in line with its ability to affect both MDSC subpopulations.

Achieving MMR is extremely important in the course of CML to avoid relapse. A recent study demonstrated that both imatinib and dasatinib treatment efficiently decreased the amount of G‐MDSC in patient with CML, but it did not found a correlation between MMR and MDSC count 31. Here, we observed a significant correlation between the number of persistent M‐MDSC with MMR value only in CML patients treated with dasatinib. MDSC are supposed to provide a favourable microenvironment in which leukaemia cells can evade host immunosurveillance and proliferate. Therefore, the elevation of M‐MDSC in the follow‐up of CML patients treated with dasatinib might indicate the delayed immune control and the higher levels of residual leukaemia cells, suggesting that M‐MDSC might have a role in predicting prognosis. Studies by Sun *et al*. 18 reported a similar observation in adult acute myeloid leukaemia (AML). The authors identified MDSC as CD33^high^CD11b^+^ HLA‐DR^low^ cells and found that their level in the high minimal residual disease (MRD) group was significantly higher than that in the middle and low MRD groups. Comparing the time to disease progression in patients with elevated pre‐treatment frequency of M‐MDSC to those with lower levels of M‐MDSC in chronic lymphocytic leukaemia (CLL), Gustafson *et al*. 32 found that patients with higher levels of M‐MDSC had a shorter time to disease progression compared to patients with lower levels. These data suggest that M‐MDSC could be predictive of poorer prognosis in different haematological malignancies.

Some patients with CML can stop imatinib without disease relapse despite the persistent presence of residual CML cells, suggesting that other endogenous factors are important for restraining CML cells even in the absence of TKI treatment. The immunosurveillance, whereby the immune cells inhibit tumour cell growth, plays a central role. The presence of relatively abundant NK cells and cytotoxic T lymphocytes (CTL) specific for CML antigens such as BCR‐ABL1 or proteinase‐3 is good candidates for predictive markers of safe TKI discontinuation 8, 9, 33. MDSC, especially monocytic subset, could be able to favour the growth of CML cells *in vivo* through impairment of immunosurveillance against LSC. Therefore, these myeloid cells could be candidate as predictive markers of relapse risk following TKI discontinuation and their evaluation before and after discontinuation of imatinib involving a large patient cohort might be important.

In addition, incubating HD‐derived monocytes with serum or exosomes from CML patients at diagnosis, we found their conversion into M‐MDSC. The CD14^+^HLA‐DR^−^ cells generated *in vitro* were capable of promoting T cell suppression, suggesting a biologically relevant crosstalk between leukaemic cells and M‐MDSC.

Overall, our data provide a positive correlation between the percentage of MDSC and MMR value in patients treated with dasatinib, suggesting their importance in clinical investigation to assess the clinical impact of treatment on patient as practice of precision medicine. Furthermore, it remains to be elucidated whether MDSC might be candidate predictive markers of relapse risk following TKI discontinuation. Moreover, our data suggest the possible development in patients with CML of a circuit primed by tumour cells that, through the release of soluble factors and exosomes, are able to expand M‐MDSC, creating an immunotolerant environment that results in T cell anergy and facilitates tumour growth.

## Conflict of interest

The authors confirm that there are no conflicts of interest.

## Supporting information


**Figure S1** Flow cytometry plots with gating strategies for the identification of MDSC cells. The figure shows a representative cytoflorimetric analysis with gates set on CD11b^+^CD33^+^CD14^−^HLA‐DR^−^ (G‐MDSC) (A) and CD14^+^HLA‐DR^−^ (M‐MDSC) (B) cell populations.
